# Event-Based Heat-Related Risk Assessment Model for South Korea Using Maximum Perceived Temperature, Wet-Bulb Globe Temperature, and Air Temperature Data

**DOI:** 10.3390/ijerph17082631

**Published:** 2020-04-11

**Authors:** Misun Kang, Kyu Rang Kim, Ju-Young Shin

**Affiliations:** Applied Meteorology Research Division, National Institute of Meteorological Sciences, Seohobuk-ro 33, Seogwipo 63568, Korea; misun0106@korea.kr (M.K.); kkr9@korea.kr (K.R.K.)

**Keywords:** heat-related mortality, heatwave, wet-bulb globe temperature, perceived temperature, maximum temperature

## Abstract

This study aimed to assess the heat-related risk (excess mortality rate) at six cities, namely, Seoul, Incheon, Daejeon, Gwangju, Daegu, and Busan, in South Korea using the daily maximum perceived temperature (PTmax), which is a physiology-based thermal comfort index, the wet-bulb globe temperature, which is meteorology-based thermal comfort index, and air temperature. Particularly, the applicability of PTmax was evaluated using excess mortality rate modeling. An event-based heat-related risk assessment model was employed for modeling the excess mortality rate. The performances of excess mortality rate models using those variables were evaluated for two data sets that were used (training data, 2000–2016) and not used (test data, 2017–2018) for the construction of the assessment models. Additionally, the excess mortality rate was separately modeled depending on regions and ages. PTmax is a good temperature indicator that can be used to model the excess mortality rate in South Korea. The application of PTmax in modeling the total mortality rate yields the best performances for the test data set, particularly for young people. From a forecasting perspective, PTmax is the most appropriate temperature indicator for assessing the heat-related excess mortality rate in South Korea.

## 1. Introduction

The adverse effects of high atmospheric temperatures on human health have been reported in many regions [1,2,3,4,5]. When people are excessively exposed to heat, the human body produces or absorbs more heat than it dissipates. This excessive exposure can lead to the failure of the thermoregulatory system of the human body [6]. Hence, this failure increases the internal temperature in the core of the body, which is considered to be heat-related stress on the human body. Finally, the temperature in the core body exceeds the threshold for optimal body comfort, performance, and health. For instance, high body temperatures can lead to the loss of salt and water through sweating, causing increases in cardiovascular diseases, such as coronary and cerebral thrombosis. Very high body temperatures also damage cellular structures and the thermoregulatory system, ultimately leading to death [7].

Human thermal comfort results from the energy balance between the human body and the environment [8]. Various factors, such as human physiology, psychology, and behavior, influence human thermal comfort. The use of thermal comfort indices, such as the perceived temperature (PT), physiological equivalent temperature, and universal thermal climate index, based on the heat exchange between the human body and its thermal environment to evaluate the biometeorological conditions has been suggested [9,10,11,12]. These thermal comfort indices have been used to investigate the relationship between heatwave events and human health and assess the heat-related risk to human health [13,14,15,16]. Several studies reported that the use of physiology-based thermal comfort indices is appropriate for the assessment of heat-related as well as cold-related health risks [17,18,19].

The heat-related risk in South Korea has been assessed in many studies depending on various factors, such as age, region, and the definition of a heatwave [20,21,22]. Although these studies were carried out under different conditions, most of them employed the maximum air temperature as a temperature indicator for the definition of a heatwave. Recently, thermal comfort indices based on meteorological variables were tested as temperature indicators for the assessment of the heat-related risk in South Korea. Heo et al. [23] evaluated the accuracy of the wet-bulb globe temperature (WBGT) in assessing the heat-related excess mortality and reported that the WBGT could be used as a temperature indicator for the heatwave in the assessment.

Global warming leads to changes in climatic conditions [24]. Particularly, the increment of air temperature increases the frequency and magnitude of heatwave [25,26]. Change in the heat-related risk may be different depending on which temperature indicator is used in heat-related risk assessment because the climate change leads to changes in temperature, humidity, radiation, and wind speed, which is related to heat-related stress. Thus, for accurate assessment of heat-related risk in climate change, the temperature indicator that can consider various parameters influencing the heat-related stress needs to be employed. Because the physiology-based thermal comfort index considers the various parameters based on thermal regulation of the human body, this index may have a consistent relationship with the heat-related mortality rate, even though the atmospheric environment is changed. Hence, the application of the PT in heat-related risk assessment would be beneficial to estimate the risk in changing the environment as well as a stationary environment. In addition, the thermal comfort index based on the physiological heat exchange of the human body has not been employed for assessing the heat-related risk in South Korea. Hence, the applicability of physiology-based thermal comfort indices must be investigated to enhance our capacity in assessing the heat-related risk in South Korea.

The aim of this study was to assess the heat-related risk in South Korea using the thermal comfort index, that is, the daily maximum PT (PTmax). Subsequently, the applicability of PTmax in the heat-related risk assessment was evaluated. For comparison, the daily maximum air temperature (Tmax) and daily maximum WBGT (WBGTmax) were used as a meteorological variable and a thermal comfort index based on meteorological variables, respectively, in assessing the heat-related risk. The event-based heat-related risk assessment model suggested by Dieter et al. [27] was employed for modeling the heat-related risk. Because many studies focused on identifying the best predictor of heat-related risk, the results of many studies might be sufficient from heat-related risk modeling or forecasting perspectives [28]. In the current study, the performances of heat-related risk assessment models using different temperature indicators for unseen data were evaluated to investigate the appropriateness of the selected temperature indicator for the heat-related risk prediction. Additionally, the mortality rate data were separately modeled depending on the region and age to identify their heat-related impacts on the health risks. The current study would expand our knowledge and improve our understanding of assessing the heat-related risk to human health in South Korea. In addition, the results were good references for the selection of temperature indicators for the development of a heat-related health warning system.

This paper is organized as follows. In Section 2, the temperature indicators and mortality and meteorological data used in this study are described, and the event-based heat-related risk assessment model is introduced. The characteristics of the risk assessment model using different temperature indicators for regions and ages are presented in Section 3. In Section 4, the applicability of PT in assessing the heat-related risk in South Korea and the limitations of the current study are discussed. Finally, the conclusions are presented in Section 5.

## 2. Materials

### 2.1. Temperature Indicators

Because the PT is an unobservable variable, it must be calculated using meteorological variables and assumed conditions. Additionally, it is often not measured at weather stations because the black globe temperature is not regarded to be a standard element. Thus, empirical and physical estimation models using other meteorological variables have been applied to obtain WBGT estimates [29,30,31]. The brief information of the employed temperature indicators is summarized in Table 1. Theoretical background and the PT and WBGT equations are briefly described in the following subsections.

#### 2.1.1. Perceived Temperature

The PT is a reference environment in which the perception of cold and/or heat is the same as that under the actual conditions [12]. Perceived heat can be computed by using the comfort equation suggested by Fanger [36], which is based on a complete heat budget model for the human body. To determine the PT, the Klima–Michel model (KMM), which is a complete heat budget model for human beings, is used for the assessment of the thermal physiology [33]. The reference person in the KMM is a male who is 35 years old, 1.75 m tall, weighs 75 kg, wears clothes, and walks at a speed of 4 km/h on flat ground. In this study, summer clothes (0.5 clo) were used because heat-related risks often occur in summer. The PT considers not only meteorological parameters, such as the air temperature, humidity, solar radiation, but also behavioral characteristics, such as activity and clothing insulation, and body measurements, such as the metabolic rate, of the reference person. Because the PT can emulate a complex system of heat perception for a human being with various parameters based on the thermal physiology mechanism, the PT may be a good parameter for the representation of the magnitude of heat-related events. The theoretical description of the PT is provided below.

The KMM is based on the heat balance equation for the human body in a two-node model given by ASHRAE [37]:

(1) $$M-W=\left(C_{\mathrm{skin}}+R_{\mathrm{skin}}+E_{\mathrm{skin}}\right)+\left(C_{\mathrm{res}}+E_{\mathrm{res}}\right)+S_{\mathrm{skin}}+S_{\mathrm{core}}$$

where M ($W\cdot m^{-2}$), W ($W\cdot m^{-2}$), C ($W\cdot m^{-2}$), R ($W\cdot m^{-2}$), and E ($W\cdot m^{-2}$) are the metabolic rate of the body, energy for mechanical work, convection, radiation, and evaporation, respectively. The metabolic rate of the body provides energy to enable the body to do mechanical work, and the remainder of the energy is released as heat (i.e., M−W). Heat transfer can occur by convection (C), radiation (R), and evaporation (E). On the right side of Equation (1), the first and second brackets represent the heat exchange based on the skin and respiration, respectively. The parameters $S_{\mathrm{skin}}$($W\cdot m^{-2}$) and $S_{\mathrm{core}}$ ($W\cdot m^{-2}$) indicate the heat storage in the skin and core compartment, respectively. Under steady-state conditions, $S_{\mathrm{skin}}$ and $S_{\mathrm{core}}$ are equal to zero due to thermoregulation. When the internal heat production equals the amount of heat exchanged with the environment and the external conditions are steady, the predicted mean vote (PMV) can be calculated as follows:

(2) $$\mathrm{PMV}=\alpha \cdot \left\{M-W-\left(C_{\mathrm{skin}}+R_{\mathrm{skin}}+E_{\mathrm{skin}}\right)-\left(C_{\mathrm{res}}+E_{\mathrm{res}}\right)\right\}=\alpha \cdot L_{th}$$

where $L_{th}$ ($W\cdot m^{-2}$) determines the thermal load. The thermal load is linearly rescaled via α(=[0.303·exp(−0.036·M)+0.0275]) to the dimensionless scale of perceived comfort. The PMV equation proposed by Gagge, Fobelets, and Berglund [32] in the KMM is employed in the PT (°C) calculation. The PT in heat stress zone (PMV > 0) is translated from the PMV using the following equation:

(3) PT=6.18·PMV+16.83

When the value of PMV is equal to or less than zero, Equation (3) should be replaced with other formulas. The detailed information and procedure for the computation of PT can be found in Staiger, Laschewski, and Grätz [12] and Parsons [38].

#### 2.1.2. Wet-Bulb Globe Temperature

WBGT (°C) is an index that is calculated as the weighted average of air temperature, natural wet bulb temperature, and black globe temperature [34] as follows:

(4) $$\mathrm{WBGT}=0.7T_{w}+0.2T_{g}+0.1T_{a}$$

where $T_{w}$ (°C), $T_{g}$ (°C), and $T_{a}$ (°C) are the natural wet bulb temperature, black globe temperature, and air temperature, respectively. In this study, the empirical estimation model suggested by the Korea meteorological administration (KMA) was used to obtain the WBGT for South Korea. This model requires $T_{a}$ and $T_{w}$, and has been known to perform well in the estimation of the WBGT in South Korea [35]. The WBGT model proposed by the KMA is as follows:

(5) $$\mathrm{WBGT}=-0.2442+0.55399T_{w}+0.45535T_{a}-0.0022T_{w}^{2}+0.00278T_{w}T_{a}$$

$T_{w}$ in Equation (5) is calculated using the equation suggested by Stull [39], as seen below.

(6) $$\begin{array}{c} T_{w}=T_{a}\tan^{-1}\left[0.151977{\left(RH+8.313659\right)}^{1/2}\right]+\tan^{-1}\left(T_{a}+RH\right)-\tan^{-1}(RH-\\ 1.67633)+0.00391838RH^{\frac{3}{2}}\tan^{-1}\left(0.023101RH\right)-4.686035, \end{array}$$

where *RH* (%) is the relative humidity.

### 2.2. Study Area and Data

**Figure 1:**
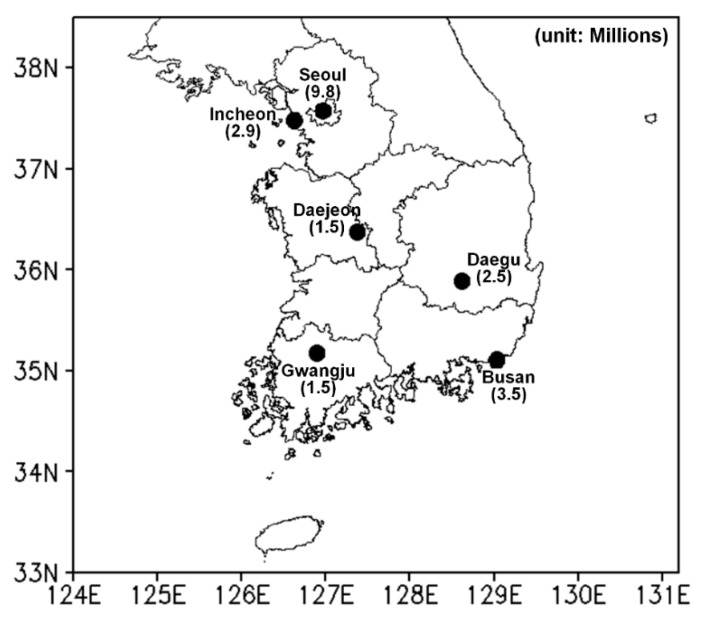
Locations and populations of the six metropolitan cities in South Korea.

In the current study, the heat-related risk was assessed by using different temperature indicators in six metropolitan cities in South Korea: Seoul, Incheon, Daejeon, Gwangju, Daegu, and Busan. The population of each city is larger than one million people. The locations and populations of all cities used in this study are described in Figure 1. The proportion of elderly people (>64 years) to the total population in Seoul, Incheon, Daejeon, Gwangju, Daegu, and Busan is 14%, 17%, 14%, 12%, 13%, and 12%, respectively. The period from 2000 to 2018 was used in the current study due to the availability of daily mortality data. Mortality and population data of each city were obtained from the MicroData Integrated Service (http://mdis.kostat.go.kr/), which is affiliated with Statistics Korea. Daily mortality and population data for South Korea are released annually by Statistics Korea. Daily mortality data, including the address, sex, age, time, place, and cause of death, are recorded in the International Statistical Classification of Diseases and Related Health Problems [40,41].

To calculate the PT and WBGT, the $T_{a}$, relative humidity (RH), dew point temperature, wind speed, cloud amount, cloud type, and geographical information regarding the instrument were obtained from meteorological stations in six cities. The hourly PT and WBGT were calculated using hourly observed meteorological parameters. Maximum hourly temperature indicator data within a day were selected for the identification of the temperature indicators: PTmax, Tmax, and WBGTmax.

### 2.3. Event-Based Heat-Related Risk Assessment Model

In this study, the concept of risk due to a hazardous event based on Dieter, Ute, Tobia, Steffen, Fred, and Christian [27] was adopted. The risk (*r*) can be determined from the hazard (*h*) and vulnerability (*v*). Hence, the risk due to a hazardous event can be represented by the following equation:

(7) r=h·v,

The hazard (h) can be quantified by using the mean magnitude rate of the hazardous event, and the vulnerability (v) is defined as the product of the exposure of the elements at risk and the sensitivity of the elements at risk exposed to the hazard. Hence, the vulnerability is given by:

(8) v=e·s,

where e and s are the exposure and sensitivity, respectively. Finally, the risk can be calculated as follows:

(9) $$r=\bar{M}\cdot e\cdot s,$$

where $\bar{M}$ is the mean magnitude rate of the hazardous event. The detailed theoretical derivation of the relationship among the risk, hazard, and vulnerability can be found in Dieter, Ute, Tobia, Steffen, Fred, and Christian [27].

The total mortality rate (p) is the total mortality ($N_{death}$) of a population (N) and consists of the excess mortality rate ($p_{h}$), which is the mortality rate related to a heatwave event, and base mortality rate ($p_{0}$), which is the mortality rate related to other reasons. This relationship is given by:

(10) $$p=\frac{N_{death}}{N}=p_{0}+p_{h}$$

In the current study, the mean heat-related excess mortality rate ($p_{h}$) over time, denoted as mean mortality rate ($10^{-6}\cdot day^{-1}$), was used to express the heat-related risk. Unfortunately, the excess and base mortality rates cannot be obtained from the observed mortality data due to complex death reasons. However, they can be estimated from the relationships between p and selected temperature indicators. In the current study, linear regression analysis was adopted to derive the relationships between p and selected temperature indicators. This method was previously employed in Buchin et al. [42] and Jänicke et al. [43] and yielded a good performance in analyzing the above-mentioned relationship. The assumed regression model is as follows:

(11) $$p=p_{0}+p_{h}=p_{0}+M\cdot \alpha +\varepsilon ,$$

where α , M, and ε are the slope of the linear regression model, magnitude of the heatwave event based on the selected temperature indicator, and the error term, respectively. The excess mortality rate can be expressed as:

(12) $$p_{h}=M\cdot \alpha$$

As shown in Equation (12), M and α can be considered as the mean magnitude rate and sensitivity in Equation (9), respectively. Because the mortality rate is used for the risk, the exposure term can only be considered after the estimation of the excess mortality rate. Thus, the exposure term is excluded from Equation (12).

A heatwave event is defined as consecutive days on which the value of the temperature indicator ($T_{x}$) exceeds a certain threshold ($T_{th}$). Hence, the magnitude of the *i*th heatwave event ($M_{i}$) can be computed as follows:

(13) $$M_{i}=\log_{10}\left[1+\sum_{j=d_{i}}^{d_{i}+D_{i}-1}\left(T_{x}\left(j\right)-T_{th}\right)\right],$$

where $d_{i}$ and $D_{i}$ indicate the starting time and duration of the *i*th heatwave event. In the current study, a heatwave event was considered to occur when $T_{x}$ exceeded $T_{th}$ on three consecutive days. Thus, $D_{i}$ is always larger than two. The mean total mortality rate ($\bar{p_{i}}$) is the total mortality rate over the period, which is adversely affected by the heatwave event. The mean total mortality rate ($\bar{p_{i}}$) of a given heatwave event is computed for the period, including the days of the event and a variable number of lag days (L). Because heat-related mortality can occur after the heatwave event, the mortality during lag days is considered when calculating $\bar{p_{i}}$. When the lag days of a previous heatwave event overlap with the days of a current heatwave event, the overlapping days are accounted for in the current heatwave event instead of the previous event. Therefore, the mean total mortality rate of the *i*th heatwave event can be given by:

(14) $$\bar{p_{i}}=\frac{1}{D_{i}+L_{i}}\sum_{j=d_{i}}^{d_{i}+D_{i}+L-1}p\left(j\right)$$

The final equation is obtained by inserting Equations (13) and (14) into Equation (11). The final equation for the heat-related risk assessment model is:

(15) $$\bar{p_{i}}=p_{0}+M_{i}\cdot \alpha +\varepsilon$$

This risk assessment model contains four free parameters: α, $p_{0}$, $T_{th}$, and L. These parameters must be estimated for the construction of the risk assessment model. Various numbers (21–50 °C for $T_{th}$ and 0–14 days for L) are tested to estimate $T_{th}$ and L. The intervals between these numbers are one. When $T_{th}$ and L are predefined, α and $p_{0}$ are estimated from the calculated $M_{i}$ and $\bar{p_{i}}$ using the ordinary least squares method. All combinations of $T_{th}$ and L are tested, and the coefficient of determination ($R^{2}$) and its *p*-values are calculated for each combination. The combination leading to the largest $R^{2}$ is selected for the heat-related risk assessment model by a given data set. The total mortality ($N_{death,i}$) during the period, including the *i*th heatwave event and lag days, can be estimated as follows:

(16) $$N_{death,i}=N_{i}\cdot \left(M_{i}\cdot \alpha +p_{0}\right)\cdot \left(D_{i}+L_{i}\right)\cdot 10^{6},$$

where $N_{i}$ is the population during the period, including the *i*th heatwave event and lag days.

Based on the risk concept presented above, heat-related risk assessment models were built for six South Korean cities (Seoul, Incheon, Daejeon, Daegu, Gwangju, and Busan). The PTmax, Tmax, and WBGTmax were used as temperature indicators. The heat-related risk assessment models were built using the observation data sets from 2000 to 2016, and their performances were evaluated using the data sets from 2017 to 2018. Young (0–64 years) and elderly (>64 years) models were separately constructed to investigate the relationships between selected temperature indicators and the excess mortality rate depending on the age. Finally, the relationships between excess mortality and magnitude of heatwave depending on the region and age in South Korea were examined using the constructed models.

The root-mean-square error (RMSE) and normalized RMSE (NRMSE) were used as evaluation criteria. Because the total mortality is the only observable variable related to the mortality rate, the performance of the risk assessment model was evaluated based on the difference between the observed and estimated total mortalities. The RMSE can be used to quantify the magnitude of the error in the risk assessment. The NRMSE represents the relative error, that is, the RMSE of the mean observed total mortality rate. Thus, the performances of different models with different free parameters can be compared using the NRMSE. The RMSE for the *j*th temperature indicator can be calculated using the following equation:

(17) $${\mathrm{RMSE}}_{j}=\sqrt{\frac{\sum_{i=1}^{n_{h}}N_{death,i}-{\hat{N}}_{death,i,j}}{n_{h}}},$$

where ${\hat{N}}_{death,i,j}$ and $n_{h}$ are the total mortality estimate of the *i*th heatwave event using the *j*th temperature indicator and number of heatwave events, respectively. The NRMSE for the *j*th temperature indicator can be calculated using the following equation:

(18) $${\mathrm{NRMSE}}_{j}=\frac{n_{h}}{\sum_{i=1}^{n_{h}}N_{death,i}}\sqrt{\frac{\sum_{i=1}^{n_{h}}N_{death,i}-{\hat{N}}_{death,i,j}}{n_{h}}}$$

## 3. Results

### 3.1. Consideration of Regions in Risk Assessment Models

**Figure 2:**
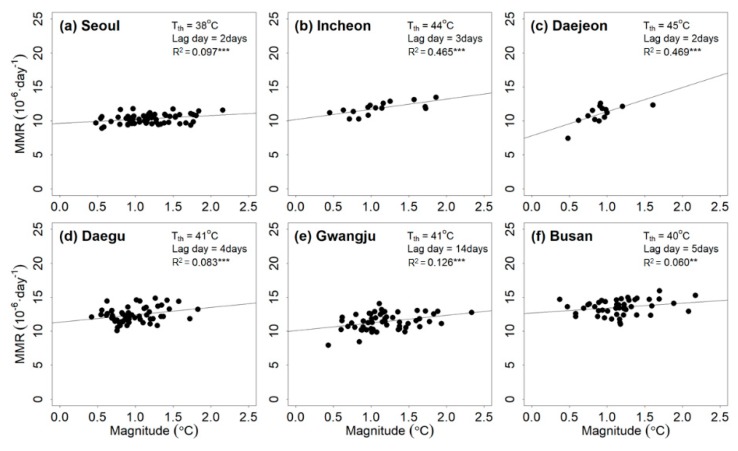
Relationship between the magnitude of the heatwave event based on maximum perceived temperature (PTmax) and mean total mortality rate (MMR) for (**a**) Seoul, (**b**) Incheon, (**c**) Daejeon, (**d**) Daegu, (**e**) Gwangju, and (**f**) Busan during the training period (2000–2016). Note that *** and ** indicate that the $R^{2}$ estimates are significant at the 95% and 90% levels, respectively; NS indicates that the $R^{2}$ estimate is insignificant based on the 85% significance level.

The relationship between PTmax and the heat-related mortality rate is shown in Figure 2. The magnitude of the heatwave event based on PTmax correlated with the mean total mortality rate. The threshold ranged from 38 to 45 °C. The lag days estimated for the six cities ranged from 2 to 14 days. Based on $R^{2}$, all regression lines were significant at the 10% level. The values ranged from 0.06 to 0.469. Large variations of these parameters indicated that the relationships between PTmax and the mean total mortality rate differed in different cities. This means that the heat-related risk with the same magnitude might differ depending on the region. With the increase in the threshold, the correlation became stronger. Because a higher threshold led to the selection of more intense heatwave events, the adverse effects of the heatwave events on the people were more apparent than those related to a lower threshold. The relation between the $R^{2}$ value and threshold overall supported this claim, although they were weakly correlated.

**Figure 3:**
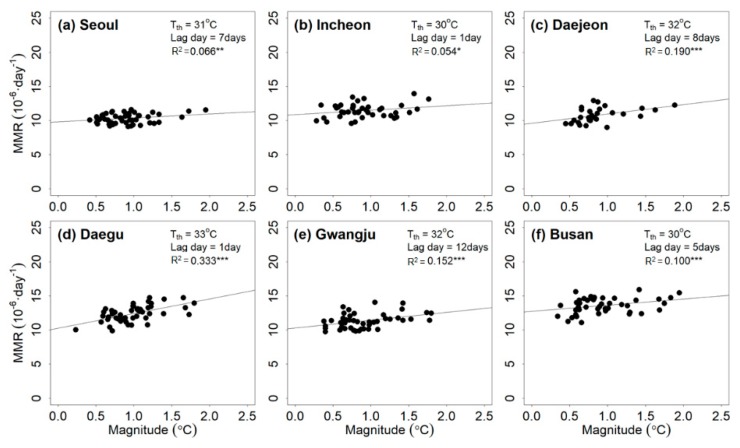
Relationship between the magnitude of the heatwave event based on maximum air temperature (Tmax) and mean total mortality rate (MMR) for (**a**) Seoul, (**b**) Incheon, (**c**) Daejeon, (**d**) Daegu, (**e**) Gwangju, and (**f**) Busan during the training period (2000–2016). Note that ***, **, and * indicate that the $R^{2}$ estimates are significant at the 95%, 90%, and 85% levels, respectively; NS indicates that the $R^{2}$ estimate is insignificant based on the 85% significance level.

Figure 3 presents the relationship between Tmax and the heat-related mortality rate for six cities. The magnitude of the heatwave event based on Tmax correlated with the mean total mortality rate. The estimated Tmax threshold ranged from 30 to 33 °C. The lag days ranged from 1 to 12 days. The $R^{2}$ values of all regression lines were significant at the 15% level and ranged from 0.054 to 0.333. Similar to the results for PTmax, the Tmax and the mean of total mortality rate were positively correlated until the threshold was reached.

**Figure 4:**
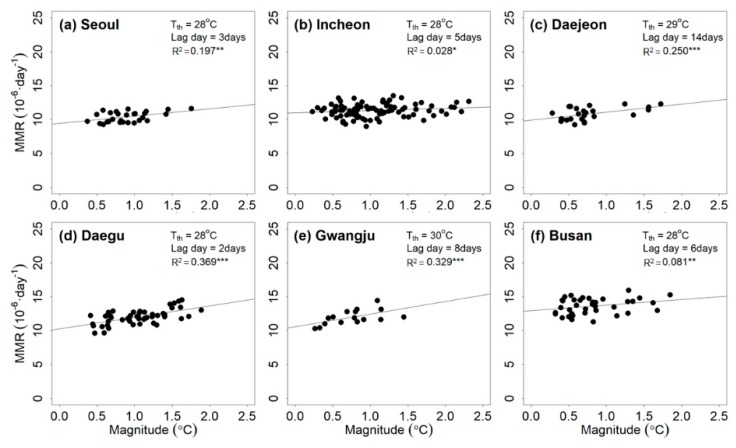
Relationship between the magnitude of the heatwave event based on maximum wet-bulb globe temperature (WBGTmax) and mean total mortality rate (MMR) for (**a**) Seoul, (**b**) Incheon, (**c**) Daejeon, (**d**) Daegu, (**e**) Gwangju, and (**f**) Busan during the training period (2000–2016). Note that ***, **, and * indicate that the $R^{2}$ estimates are significant at the 95%, 90%, and 85% levels, respectively; NS indicates that the $R^{2}$ estimate is insignificant based on the 85% significance level.

Figure 4 presents the relationship between WBGTmax and the heat-related mortality rate for the six cities. The magnitude of the heatwave event based on WBGTmax correlated with the mean total mortality rate. The WBGTmax threshold ranged from 28 to 30 °C. The lag days ranged from 3 to 14 days. The $R^{2}$ values of all regression lines were significant at the 15% level and varied from 0.028 to 0.339.

The estimates of the four free parameters, $R^{2}$, and annual mean excess mortality rate (MEMR) for the heat-related risk assessment models depending on the regional characteristics are presented in Table 2.

The temperature indicator leading to the largest $R^{2}$ differed depending on the city. The use of PTmax for the modeling of the excess mortality rate yielded the largest $R^{2}$ among the three temperature indicators in Incheon and Daejeon. The $R^{2}$ values were 0.465 and 0.469, respectively, and were significant at the 5% level. The application of Tmax led to the best performance based on the $R^{2}$ estimate when modeling the excess mortality rate in Busan. The $R^{2}$ value was 0.100 and was significant at the 5% level. WGBTmax yielded the largest $R^{2}$ in Seoul, Daegu, and Gwangju. The $R^{2}$ values were 0.197, 0.369, and 0.329, respectively, and significant at the 5% level.

**Figure 5:**
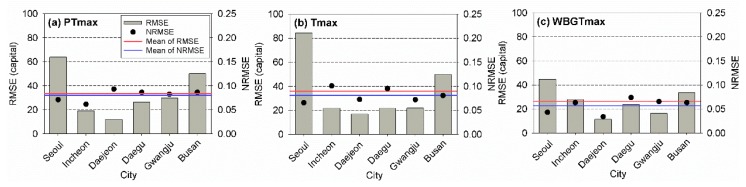
Root-mean-square error (RMSE) and normalized RMSE of the observed and estimated annual total mortalities for (**a**) PTmax, (**b**) Tmax, and (**c**) WBGTmax for the six cities during the training period (2000–2016).

To evaluate the performances of the heat-related risk assessment models using different temperature indicators considering different regions in South Korea, the RMSE and NRMSE values of the observed and estimated total mortality rates were calculated for all employed cities. The RMSE and NRMSE values for the six cities during the training period are shown in Figure 5. Overall, the use of WBGTmax led to the best performances based on the RMSE and NRMSE. The best temperature indicator for the heat-related risk assessment might be the WBGTmax, except for Incheon, where PTmax yielded the smallest RMSE and NRMSE values.

**Figure 6:**
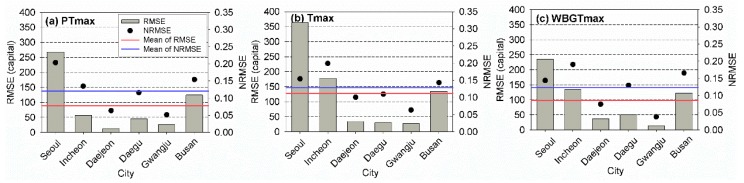
Root-mean-square error (RMSE) and normalized RMSE of the observed and estimated annual total mortalities for (**a**) PTmax, (**b**) Tmax, and (**c**) WBGTmax for the six cities during the test period (2017–2018).

These results did not represent the performances of the models for unseen data sets because of the data sets that were used to build the models. Thus, to evaluate the performances of the models in predicting the heat-related risk, the RMSE and NRMSE values of the test period were calculated. The results are presented in Figure 6. The numbers of the event for test period were eight for PTmax in Seoul, two for PTmax in Incheon, seven for PTmax in Daejeon, nine for PTmax in Daegu, nine for PTmax in Gwangju, seven for PTmax in Busan, four for Tmax in Seoul, two for Tmax in Incheon, six for Tmax in Deajeon, six for Tmax in Deagu, six for Tmax in Gwangju, four for Tmax in Busan, five for WBGTmax in Seoul, eight for WBGTmax in Incheon, five for WBGTmax in Deajoen, eight for WBGTmax in Deagu, six for WBGTmax in Gwangju, and six for WBGTmax in Busan, respectively. The RMSE and NRMSE values determined for the test period were much larger than those of the training period. If the relationship between the mortality and heatwave event was constant, the difference in the evaluation criteria between the two periods might be small. Large differences might indicate that the temporal variability in the relationship between the mortality and heatwave was large in South Korea. In contrast to the results obtained for the training period, the use of PTmax for the heat-related risk assessment overall led to the best performance based on the RMSE and NRMSE values for the test period. The performances of the models using WBGTmax were better than those obtained using Tmax. Based on the NRMSE, models using PTmax led to the best performances in Incheon, Daejeon, Daegu, and Busan. The application of WBGTmax in heat-related risk assessment models yielded the smallest NRMSEs in Seoul and Gwangju. Thus, PTmax and WBGTmax were appropriate temperature indicators that could be used for the assessment of the heat-related risk in South Korea.

### 3.2. Consideration of the Age in Risk Assessment Models

To investigate the relationship between the selected temperature indicator and excess mortality rate of young and elderly people, the mortality rates were classified into young and elderly categories. To focus on the heat-related impact of different ages, the data sets for the six cities were integrated into the constructions of event-based heat-related risk assessment models. The estimates of the four free parameters, $R^{2}$, and annual MEMR depending on the age during the training period are presented in Table 3. For each temperature indicator, the threshold estimates were identical for different ages (i.e., young and elderly), while the estimates of other free parameters (e.g., lag day, slope, and base mortality rate) differed. The slopes obtained for the different temperature indicators were similar within an age category. For instance, the slopes determined for the young and elderly people ranged from 0.16 to 0.2 and from 2.24 to 3.98, respectively. The slopes determined for elderly people were higher than those determined for young people. The values of the slopes for elderly people were ~16 times those of young people. This means that elderly people are more vulnerable to heat than young people in South Korea. The base mortalities estimated for each age group were similar. The base mortality estimates determined for the young and elderly people ranged from 4.31 to 4.33 and from 84.28 to 86.09, respectively. This indicated that heat-related risk assessment models with different temperature indicators might yield reliable estimates of the base mortality rate. Based on $r^{2}$, Tmax was the most appropriate temperature indicator for the constructed model considering the age.

**Figure 7:**
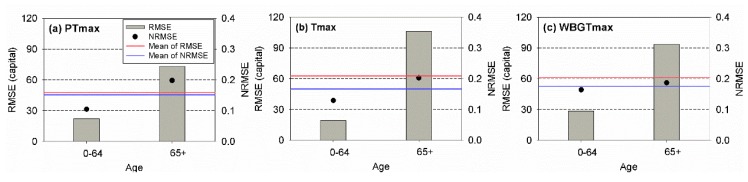
Root-mean-square error (RMSE) and normalized RMSE of the observed and estimated annual total mortalities for (**a**) PTmax, (**b**) Tmax, and (**c**) WBGTmax for young (0–64) and elderly (64+) people for the training period (2000–2016).

The RMSE and NRMSE values determined for the training period for different ages are presented in Figure 7. Overall, the risk assessment models using PTmax led to the best performances based on the RMSEs and NRMSEs for each age category. For young people, the use of Tmax and PTmax yielded the smallest RMSE and NRMSE, respectively. The application of PTmax and WBGTmax yielded the smallest RMSE and NRMSE for elderly people, respectively. Although the models using PTmax seemed to be the best based on the results, the difference in the performances of the employed models was small.

**Figure 8:**
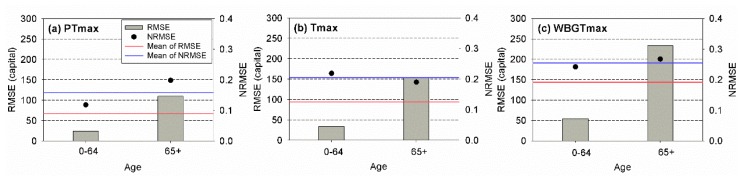
Root-mean-square error (RMSE) and normalized RMSE of the observed and estimated annual total mortalities for (**a**) PTmax, (**b**) Tmax, and (**c**) WBGTmax for young (0–64) and elderly (64+) people for the test period (2017–2018).

To evaluate the performances of the models in predicting the heat-related risk for unseen data sets, the RMSE and NRMSE values of different temperature indicators during the test period were calculated for different age groups. The results are presented in Figure 8. The numbers of the event for the test period were nine for PTmax, nine for Tmax, and seven for WBGTmax, respectively. The patterns of the RMSE and NRMSE obtained for the test period were similar to those of the training period, although the RMSE and NRSME values determined for the test period were much larger than those of the other period. Overall, risk assessment models using PTmax led to the best performance based on the RMSE and NRMSE for each age group. The models using PTmax yielded the smallest RMSEs among the employed models for all age groups. In addition, the model using PTmax for young people yielded the smallest NRMSE. For elderly people, the model using Tmax led to the best performance based on the NRSME. These results indicated that PTmax should be applied for the assessment of the heat-related risk in South Korea, particularly for young people.

### 3.3. Consideration of the Region and Age in Risk Assessment Models

The mortality rates were classified into twelve groups considering regions (Seoul, Incheon, Daejeon, Daegu, Gwangju, and Busan) and ages (young and elderly people) to examine the relationship between the heat-related risk and heatwave depending on regions and ages in detail. The estimates of the four free parameters, $R^{2}$, and annual MEMR during the training period depending on regions and ages are listed in Table 4.

Although the free parameter estimates varied with the regions and ages, regional characteristics had a larger impact on the free model parameters. For instance, the thresholds of the models using PTmax for young and elderly people ranged from 33–45 °C and from 32–41 °C, respectively. The lag day and slope estimates seemed to be the most variable parameters in the constructed model. For example, the lag days for the elderly people ranged from 1–14 days for PTmax, 1–14 days for Tmax, and 0–14 days for WBGTmax. The lag day estimates covered the whole range of tested lag days. In addition, the slope estimates based on Tmax for elderly people in Incheon and Busan were 14.03 and 1.7, respectively. The slope value determined for Incheon was nine times that obtained for Busan. The $r^{2}$ values of the models for young people were higher than those calculated for elderly people. The $r^{2}$ values of the models using PTmax, Tmax, and WBGTmax were insignificant in four, five, and six models with a 15% significance level, respectively. Based on $R^{2}$, PTmax was the most appropriate temperature indicator considering regions and ages.

**Figure 9:**
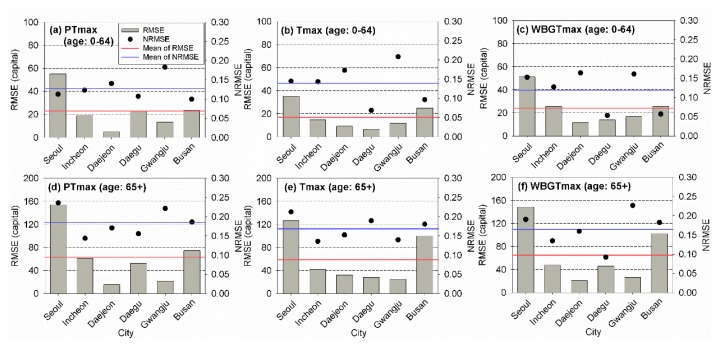
Root-mean-square error (RMSE) and normalized RMSE of the observed and estimated annual total mortalities for young people with (**a**) PTmax, (**b**) Tmax, and (**c**) WBGTmax and elderly people with (**d**) PTmax, (**e**) Tmax, and (**f**) WBGTmax for the six cities and young (0–64) and elderly (64+) people for the training period (2000–2016).

The RMSE and NRMSE values based on different temperature indicators for different regions and ages during the training period are presented in Figure 9. Heat-related risk assessment models using PTmax and Tmax for the two age categories overall showed the best performances based on the RMSE and NRMSE values. For young people, the application of PTmax yielded the smallest RMSE in two cities and the smallest NRMSE in four cities, respectively. The models using Tmax yielded the smallest RMSE and NRMSE values in three cities for elderly people. Based on the NRMSE, WBGTmax should be applied as a temperature indicator in the assessment model.

**Figure 10:**
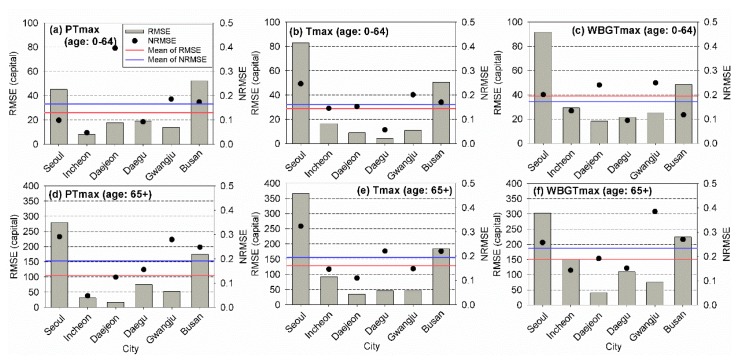
Root-mean-square error (RMSE) and normalized RMSE of the observed and estimated annual total mortalities young people with (**a**) PTmax, (**b**) Tmax, and (**c**) WBGTmax and elderly people with (**d**) PTmax, (**e**) Tmax, and (**f**) WBGTmax for the six cities and young (0–64) and elderly (64+) people for the test period (2017–2018).

The RMSE and NRMSE values based on different temperature indicators for different regions and ages for the test period are presented in Figure 10. In contrast to the results of the heat-related risk assessment models for different regions and ages during the training period, the models using PTmax overall led to the best performances based on the RMSE and NRMSE. For young people, the application of PTmax yielded the smallest RMSE in four cities and the smallest NRMSE in three cities, respectively. The models using PTmax yielded the smallest RMSE in four cities and the smallest NRMSE in two cities for elderly people, respectively. The application of Tmax yielded the smallest RMSE and NRMSE values in two cities for young people. For elderly people, the models using Tmax yielded the smallest RMSE in two cities and the smallest NRMSE in three cities, respectively. Based on the results for the test period, heat-related risk assessment models using WBGTmax yielded the worst performances among the models for South Korea using temperature indicators.

## 4. Discussion

The results of this study were used to determine the applicability of PTmax for heat-related risk assessment. The results showed that PTmax was an appropriate temperature indicator that could be used to assess the heat-related risk in South Korea. Overall, PTmax was the most appropriate temperature indicator for the assessment based on the results obtained for all cases during the test period. The heat-related risk assessment model using PTmax also showed good performance during the training period. During the training period, PTmax might be the most appropriate temperature indicator based on the RMSE and NRMSE, except for regional modeling. In contrast to the RMSE and NRMSE obtained for the training period, PTmax was the most appropriate temperature indicator in one case considering regions and ages based on $R^{2}$. Based on the $R^{2}$ value for the training period, the most appropriate temperature indicators differed depending on the cases.

From a forecasting perspective regarding heat-related risk assessment, the consistent performance of the model in the future, that is, for unseen data, is crucial. Thus, a model that consistently yields a good performance is considered to be a good model for the assessment of the heat-related health risk. The results obtained for the test period indicated the consistent performance of the employed risk assessment models. As mentioned above, heat-related risk assessment models using PTmax showed good performances in the prediction of the total mortality in all cases during the test period. These results supported the future application of PTmax, which yielded a good precision in assessing the heat-related risk. Therefore, PTmax could be applied as a temperature indicator for the assessment of the heat-related health risk in South Korea.

The high consistency of the performance of the heat-related risk assessment model indicated that the model might properly respond to heat-related mortality variability. Due to the complex mechanism of mortality, predicting the heat-related mortality is a difficult task [44,45,46,47]. The possible reason for the adequate response of PTmax to varying heat-related mortalities is that the PT is based on the heat budget model (namely KMM) for human beings. Because Tmax and WBGTmax do not consider physiological characteristics of people, the heat-related risk assessment model may be overfitted to the employed data sets, where one or several parameters illustrate meteorological conditions. On the other hand, PTmax successfully represents the magnitude of heat stress on people because it accounts for the interactions among meteorology, environment, and thermal physiology. Thus, the application of PTmax in the heat-related risk assessment model may successfully reveal the relationship between the heat-related risk and magnitude of the heatwave event. For instance, the heat-related risk assessment model using PTmax for young people showed the best performance based on the results presented in Figure 7 and Figure 8. The performance was better than that for elderly people. Because the reference person in the KMM, which is the heat budget model used for the PT, was 35 years old, the model using PT might yield good performance for young people. This result supported the claim that PT successfully revealed the relationship between the excess mortality rate and magnitude of the heatwave event due to the use of the heat budget model.

In the current study, additional meteorological variables, other than the exclusion of Tmax, WBGTmax, and PTmax, were not considered when modeling the mortality rate. A number of factors affect the mortality rate [48,49,50]. To clearly extract the heat-related impact on the mortality rate, the effects of other factors on the mortality rate should be removed [51]. Thus, only the overall relationship between the mortality rate and magnitude of the heatwave event could be identified in the current study. The current study focused on identifying the applicability of PTmax and comparing the use of PTmax, WBGTmax, and Tmax for the assessment of the heat-related risk in South Korea. The identification of the detailed relationship between the magnitude of heatwave and mortality rate was beyond the scope of the current study. Therefore, the performances of the heat-related risk assessment models using the employed temperature indicators presented in the current study might change when additional variables, such as air pollution and relative humidity, are included in the model.

To obtain the PT, a number of parameters, such as the air temperature, dew point temperature, relative humidity, wind speed, cloud amount, cloud type, and geographical information, is required. Data on the cloud amount and cloud type are often not observed at weather stations. Thus, the PT cannot be adopted for the heat-related risk assessment in many regions. In the current study, the performances of the risk assessment models using Tmax and WBGTmax were comparable with those using PTmax, although the models using PTmax showed a slightly better performance. When meteorological variables used for the PT are unavailable at a given location, Tmax and WBGTmax are good alternatives for the assessment of the heat-related risk in South Korea. Based on the results of the current study, the PT would be the best temperature indicator to express heat-related stress for Koreans. To enlarge our capacity to assess heat-related stress and risk for Korean, many meteorological variables, such as temperature, wind speed, relative humidity, shortwave radiation, and longwave radiation, should be recorded with a high network density. Installing a compact weather station would be a good manner to obtain this information, particularly in urban areas [52].

The spatial heterogeneity of heat-related stress within the employed cities is largely based on the results of the current study, particularly to the lag day and slope estimates. There is a number of factors influencing the lag day, which represents how long the heatwave impacts on mortality, and slope estimate, which represents sensitivity to heat-related stress. Although the main driver to make the large spatial heterogeneity may be the climate condition, many factors, such as climatic conditions, altitude, urbanization, facility, and public health, are associated with the spatial heterogeneity of the heat-related risk [53]. Particularly, the local impacts, such as urbanization and facility, may be critical factors for determining the lag day and slope estimates [54]. In South Korea, Hong et al. [55] reported that the urban heat island in Seoul was correlated with socio-economic development, and this heat island would aggravate heatwave events. The study areas in the current study were metropolitan cities in South Korea and had different characteristics of urbanization and facility. Thus, these local impacts may lead to the large spatial heterogeneity of heat-related stress in South Korea.

Based on the results obtained for cases considering the age, the use of PTmax in the heat-related risk assessment model led to a good performance. The possible reason for this is that the PT in this study was based on the KMM and thus the age of 35 years. The sensitivity to the heat-related stress of elderly people differs from that of young people [56,57]. To explain the heat stress on elderly people, the PT should be tuned for this age group. Matzarakis et al. [58] introduced the Klima–Michel senior model (KMSM) with a changed age, weight, and activity. The reference person was 75 years old, 1.75 m tall, weighed 70 kg and performed an activity equivalent to walking at a speed of 1 km/h. For elderly people, the PT should be calculated using the KMSM and applied for the assessment of the heat-related risk in South Korea. The parameters in the KMM were evaluated for the Korean people, and it was found that the parameters used in the KMM were applicable for young Koreans because the difference in metabolic rates from the two parameter sets was small. However, the parameters in the KMSM have not been evaluated for the elderly Koreans. The feasibility of the parameters in the KMSM should be evaluated before the application of KMSM into PT calculation for elderly Koreans. Thus, the application of KMSM in the PT model remains for further study. In addition, a methodology assessing the heat-related risk by concurrently considering two different types of PTs should be developed to improve our understanding of the heat-related risk.

## 5. Conclusions

In the current study, the heat-related risk in South Korea was assessed using PTmax, WBGTmax, and Tmax. The relationships between the heat-related risk and heatwave depending on different temperature indicators were examined for different regions (Seoul, Incheon, Daejeon, Gwangju, Daegu, and Busan) and ages (young and elderly) in South Korea. The applicability of PTmax in assessing the heat-related risk in South Korea was investigated using the results of the current study, and the performances of the heat-related risk assessment models using PTmax were compared with those obtained using WBGTmax and Tmax. 

The PTmax was considered to be a good temperature indicator that could be used for the assessment of the heat-related risk in South Korea. Risk assessment models using PTmax showed the best performances based on the RMSE and NRMSE values and could be used for future data sets. Particularly, the model using PTmax for young people yielded the best performance, which might be due to the fact that the reference person in the heat budget model used to determine the PT was 35 years old. This would explain why the PT appropriately revealed the relationship between the magnitude of heatwave events and the heat-related mortality rate and responded to varying heat-related mortalities. The relationship between the heatwave event and heat-related mortality rate largely varied depending on the regions in South Korea, while that depending on different ages was relatively consistent. Regional characteristics of the heat-related impact on the health risk should be considered to obtain reliable results when assessing the heat-related risk in South Korea.

## Figures and Tables

**Table 1:** Information of the employed temperature indicators.

Index (Unit)	Perceived Temperature (°C)	Wet Bulb Globe Temperature (°C)	Air Temperature (°C)
Type	Rationale	Direct	Direct
Measured of derived	Derived	Derived	Measured
Thermophysiological model	Klima–Michel model (KMM), parameterizations derived from a two-node model [32]	NA	NA
The measure of assessment scale	Thermal perception; thermophysiological stress, directly linked to PMV-scale	NA	NA
Input variables	$T_{a}$, *RH*, wind speed, mean radiant temperature, M	$T_{a}$, *RH*	$T_{a}$
Ease of interpretation	Complex	Moderate	Simple
Reference	Jendritzky et al. [33] Staiger, Laschewski, and Grätz [12]	Yaglou and Minard [34] Lee et al. [35]	NA

PMV, $T_{a}$ and RH indicate predicted mean vote, air temperature and relative humidity.

**Table 2:** Estimated free parameters, coefficient of determination, and annual mean excess mortality rate (MEMR) for the fitted heat-related risk assessment model depending on the regions during the training period (2000–2016).

Indicator	City	$T_{th}$ (°C)	L (Day)	α (10^−6^ ·Day^−1^ °C)	$p_{0}$ (10^−6^ ·Day^−1^)	$R^{2}$	Annual MEMR (10^−6^ ·Year^−1^)	The Number of Events
PTmax	Seoul	38	2	0.59	9.63	0.097 ***	23.27	58
	Incheon	44	3	1.51	10.18	0.465 ***	16.41	16
	Daejeon	45	2	3.56	7.79	0.469 ***	22.36	14
	Daegu	41	4	1.07	11.35	0.083 ***	37.66	57
	Gwangju	41	14	1.13	10.08	0.126 ***	101.08	54
	Busan	40	5	0.72	12.69	0.060 **	30.08	46
Tmax	Seoul	31	7	0.57	9.79	0.066 **	19.89	48
	Incheon	30	1	0.65	10.88	0.054 *	11.70	41
	Daejeon	32	8	1.38	9.58	0.190 ***	32.91	28
	Daegu	33	1	2.14	10.27	0.333 ***	51.30	49
	Gwangju	32	12	1.15	10.28	0.152 ***	57.19	45
	Busan	30	5	0.90	12.75	0.100 ***	33.10	44
WBGTmax	Seoul	28	3	1.08	9.43	0.197 ***	18.34	28
	Incheon	25	5	0.33	11.00	0.028 *	32.01	89
	Daejeon	29	14	1.18	9.93	0.250 ***	31.48	25
	Daegu	28	2	1.69	10.26	0.369 ***	65.33	50
	Gwangju	30	8	1.86	10.57	0.329 ***	21.44	16
	Busan	28	3	0.81	12.95	0.081 ^**^	20.10	37

Note that ***, **, and * indicate that the $R^{2}$ estimates are significant at the 95%, 90%, and 85% levels, respectively; NS indicates that the $R^{2}$ estimate is insignificant based on the 85% significance level.

**Table 3:** Estimated free parameters, coefficient of determination, and annual mean excess mortality rate (MEMR) for the fitted heat-related risk assessment model depending on the age for the training period (2000–2016).

Indicator	Age	$T_{th}$ (°C)	L (Day)	α (10^−6^·Day^−1^ °C)	$p_{0}$ (10^−6^·Day^−1^)	$R^{2}$	Annual MEMR (10^−6^ ·Year^−1^)	The Number of Events
PTmax	0–64	38	7	0.16	4.31	0.031 ^NS^	12.05	64
	65+	38	6	2.56	84.25	0.010 ^NS^	184.5	64
Tmax	0–64	29	2	0.20	4.33	0.053 ***	13.41	83
	65+	29	13	3.98	85.09	0.027 *	479.22	83
WBGTmax	0–64	25	0	0.16	4.33	0.045 **	14.58	80
	65+	25	7	2.24	86.09	0.014 ^NS^	287.23	80

Note that ***, **, and * indicate that the $R^{2}$ estimates are significant at the 95%, 90%, and 85% levels, respectively; NS indicates that the $R^{2}$ estimate is insignificant based on the 85% significance level.

**Table 4:** Free parameters, coefficient of determination, and annual mean excess mortality rate (MEMR) of the fitted heat-related risk assessment model depending on regions and ages for the training period (2000–2016).

Indicator	City	Age	$T_{th}$ (°C)	L (Day)	α (10^−6^·Day^−1^·°C)	$p_{0}$ (10^−6^·Day^−1^)	$R^{2}$	Annual MEMR (10^−6^·Year^−1^)	The Number of Events
PTmax	Seoul	0–64	36	7	0.25	3.60	0.049 ***	24.45	89
	Incheon		36	7	0.11	4.43	0.006 ^NS^	39.42	89
	Daejeon		45	1	1.65	2.20	0.413 ***	45.08	14
	Daegu		36	12	0.26	4.31	0.033 **	11.53	102
	Gwangju		33	4	0.29	3.75	0.044 ***	59.74	150
	Busan		36	6	0.38	4.90	0.106 ***	9.11	86
	Seoul	65+	36	3	7.26	68.97	0.045 ***	520.67	89
	Incheon		38	14	3.92	86.61	0.013 ^NS^	411.38	61
	Daejeon		41	2	3.88	79.60	0.007 ^NS^	148.29	57
	Daegu		33	8	3.06	83.72	0.014 ^NS^	628.42	121
	Gwangju		35	1	4.63	84.86	0.022 **	572.93	132
	Busan		32	4	4.01	83.23	0.025 **	600.03	114
Tmax	Seoul	0–64	30	1	0.25	3.74	0.037 **	8.71	78
	Incheon		26	1	0.19	4.39	0.022 **	21.82	149
	Daejeon		28	2	0.33	3.70	0.038 ***	33.75	132
	Daegu		35	3	0.54	4.29	0.155 *	4.07	15
	Gwangju		28	2	0.26	3.91	0.026 **	33.68	145
	Busan		27	5	0.16	5.27	0.026 *	17.80	90
	Seoul	65+	30	3	5.29	74.40	0.018 ^NS^	230.24	78
	Incheon		31	10	14.03	85.09	0.095 ^NS^	269.04	22
	Daejeon		29	14	6.31	80.63	0.030 **	972.44	110
	Daegu		33	1	8.22	80.75	0.023 ^NS^	197.06	49
	Gwangju		31	9	4.22	86.38	0.018 ^NS^	298.85	64
	Busan		26	7	1.70	89.11	0.066 ^NS^	273.97	106
WBGTmax	Seoul	0–64	25	0	0.12	3.81	0.019 ^NS^	8.91	94
	Incheon		23	3	0.06	4.50	0.068 ***	10.23	94
	Daejeon		26	3	0.46	3.51	0.096 ***	35.35	73
	Daegu		26	14	0.24	4.45	0.042 **	35.58	77
	Gwangju		23	3	0.29	3.75	0.068 ***	62.89	101
	Busan		25	13	0.18	5.24	0.036 *	25.62	66
	Seoul	65+	26	3	0.57	79.90	0.016 ^NS^	32.94	94
	Incheon		24	7	1.45	91.31	0.005 ^NS^	222.42	100
	Daejeon		26	3	2.32	83.88	0.005 ^NS^	179.98	73
	Daegu		25	14	3.71	83.43	0.031 **	719.90	94
	Gwangju		25	0	3.33	88.92	0.012 ^NS^	362.73	100
	Busan		24	4	2.30	87.47	0.010 ^NS^	304.89	76

Note that ***, **, and * indicate that the $R^{2}$ estimates are significant at the 95%, 90%, and 85% levels, respectively; NS indicates that the $R^{2}$ estimate is insignificant based on the 85% significance level.
